# Interactive gerontechnology for fall prevention in the elderly: a descriptive study

**DOI:** 10.1590/0034-7167-2022-0739

**Published:** 2023-05-08

**Authors:** Juliana Cunha Maia, Jamylle Lucas Diniz, Caroline Ribeiro de Sousa, Francisco Gerlai Lima Oliveira, Brenda Pinheiro Evangelista, Janaína Fonseca Victor Coutinho, Marília Braga Marques, Rachel Gabriel Bastos Barbosa

**Affiliations:** I Universidade Federal do Ceará. Fortaleza, Ceará, Brazil

**Keywords:** Nursing, Elderly, Accidents by Falls, Educational Technology, Health Promotion, Enfermería, Anciano, Accidentes por Caídas, Tecnología Educacional, Promoción de la Salud, Enfermagem, Idoso, Acidentes por Quedas, Tecnologia Educacional, Promoção da Saúde

## Abstract

**Objectives:**

to develop interactive gerontechnology for the prevention of falls in the elderly at home.

**Methods:**

an exploratory and descriptive study that consisted of gerontechnology development and evaluation by experts and the target audience. For evaluation, researchers used the Agreement Index (AI), considering values greater than 80%.

**Results:**

the three-dimensional virtual scale model was elaborated through the SketchUp program, with the distribution of rooms and floors, constituting Prototype 1 (P1). Fifty-four judges evaluated the P1, and all presented agreement above the established, with a minimum AI of 88% and a maximum of 100%, producing Prototype 2 (P2). Thirty elderly participants from a philanthropic institution evaluated Prototype 2. On all items, AI ranged from 83% a 100%, resulting in the final version.

**Conclusions:**

the product of this research reveals itself as an innovative and scientifically based tool aimed at preventing falls in the elderly.

## RESUMO

**Objetivos:**

desenvolver gerontecnologia interativa para prevenção de quedas em idosos no domicílio.

**Métodos:**

estudo exploratório e descritivo que consistiu no desenvolvimento da gerontecnologia e avaliação por especialistas e pelo público-alvo. Para avaliação, utilizou-se o Índice de Concordância (IC), considerando valores maiores que 80%.

**Resultados:**

elaborou-se a maquete tridimensional virtual por meio do programa SketchUp, com a distribuição dos cômodos e andares, constituindo o Protótipo 1 (P1). O P1 foi avaliado por 54 juízes, e todos apresentaram concordância acima do estabelecido, com IC mínimo de 88% e máximo de 100%, produzindo o Protótipo 2 (P2). Este foi submetido à avaliação por 30 idosos participantes de instituição filantrópica. Em todos os itens, o IC variou de 83% a 100%, resultando na versão final.

**Conclusões:**

o produto desta pesquisa se revela como ferramenta inovadora e cientificamente fundamentada visando à prevenção de quedas em pessoas idosas.

## RESUMEN

**Objetivos:**

desarrollar gerontecnología interactiva para prevención de caídas en ancianos en el domicilio.

**Métodos:**

estudio exploratorio y descriptivo que consistió en el desarrollo de la gerontecnología y evaluación por especialistas y por público objetivo. Para evaluación, utilizado el Índice de Concordancia (IC), considerando valores mayores que 80%.

**Resultados:**

elaborado la maqueta tridimensional virtual mediante el programa SketchUp, con la distribución de las habitaciones y pisos, constituyendo el Prototipo 1 (P1). El P1 fue evaluado por 54 jueces, y todos presentaron concordancia arriba del establecido, con IC mínimo de 88% y máximo de 100%, produciendo el Prototipo 2 (P2). Esto fue sometido a la evaluación por 30 ancianos participantes de institución filantrópica. En todos los ítems, el IC varió de 83% a 100%, resultando en la versión final.

**Conclusiones:**

el producto de esta investigación se revela como herramienta innovadora y científicamente fundamentada objetivando a la prevención de caídas en personas ancianas.

## INTRODUCTION

Accidents due to falls are one of the principal causes of hospitalization and deaths and contribute to the worsening of the health conditions of the elderly population^(1)^. Falls are caused by the unintentional displacement of the body during movement when there is no support and are determined by multifactorial circumstances that compromise stability^(2-3)^. The factors that predispose to its occurrence comprise intrinsic conditions (a natural or pathological process of aging^(4)^ and extrinsic factors, such as the environment where the elderly move, which is the most frequent cause of falls^(2,5)^. Improper environmental conditions constitute potential hazards for falls, such as inadequate lighting, slippery surfaces, environments with obstacles, and loose carpets^(6)^.

The World Health Organization estimates at least one fall in the population over the age of 65 per year. In Brazil, the rate of falls in the elderly varies between 10.7% and 59.3%. Interventions for fall prevention are more directed to extrinsic factors since, in most cases, they can be modified^(7)^. Fall prevention strategies among the elderly at home need to be based on health education^(8)^ with the help of educational resources, professional training, and the creation of safe environments^(9)^. Among these strategies, the use of educational resources stands out. Such resources need facilitate the discussion on the risks of falls at home and address the individual specificities and benefits in adopting behavioral changes^(10)^. When these resources are developed for the elderly population, they are called gerontechnologies.

Gerontechnology is characterized by the development of techniques, products, and services based on aspects of the aging process to improve the quality of life of the elderly, in the support of daily activities, in the prevention of diseases and illnesses, and in the promotion of health^(11)^. Its use allows a differentiated look at the care process, providing innovation and improvement of strategies that enable changes in the daily practices of older people^(12-15)^.

Among the gerontechnologies, those that have three-dimensionality stand out. The three-dimensional visualization for training and care of the elderly can be effective in preventing falls^(16-17)^. Three-dimensionality provides visual quality that favors the conceptualization of information with intervention by the health professional, providing the representation of reality^(18)^. Thus, this study seeks to contribute to the elderly person assuming the role of the active subject in the construction and consolidation of fall accident prevention practices.

## OBJECTIVES

To develop interactive three-dimensional gerontechnology for fall prevention of the elderly at home.

## METHODS

### Ethical aspects

The research met the national standards of ethics in research with human beings and was approved by the Research Ethics Committee of the Federal University of Ceará.

### Design, period, and place of study

An exploratory and descriptive study that included the development and evaluation of a three-dimensional educational gerontechnology of scale model-type with a focus on education and health promotion for the prevention of falls in the elderly at home. It consisted of the development of gerontechnology, evaluation by experts, and evaluation by the target audience. The study was guided by the instrument Strengthening the Reporting of Observational Studies in Epidemiology (STROBE)^(19)^.

The research was conducted in Fortaleza, the state of Ceará, Brazil. The creation of the educational scale model and the invitations sent by the expert judges for evaluation of gerontechnology were conducted at the premises of the Nursing Department of the Federal University of Ceará (UFC), in Fortaleza, in October 2019.

The evaluation by the target audience took place in December 2019 in a non-profit association that owns a Social Assistance Program for the elderly and aims to promote human development, the rescue of citizenship, and the search for quality of life. The institution has the participation of more than 200 registered seniors and has ample physical space for the execution of educational activities. It is a field of actions, strategies, and research linked to the Group for Research, Teaching, and Extension in Elderly Health of the Department of Nursing of the Federal University of Ceará (GEPESI).

### Population or sample; criteria of inclusion and exclusion

Fifty-four specialists and thirty elderly evaluated the educational gerontechnology. The study selected judges by intentional non-probabilistic sampling. Judges needed to meet the following criteria to compose the panel: health professionals, without age and gender restrictions, who work in the academic and/or practice area, with extensive capacity acquired by a high degree of knowledge, skill, and extensive experience with Gerontological studies, or who already had experience in development studies and evaluation of educational technologies. This information was consulted on the Lattes platform.

The evaluation of gerontechnology by the target audience included the participation of 30 seniors, who were invited as they attended the institution. The study included seniors aged 60 years or more, cognitively preserved, verified through the application of the Mini-Mental State Examination (MMSE), which excluded those with visual and/or hearing impairment and other conditions that make communication impossible. The cutoff point of the MMSE was established according to the level of education, considering 13 for illiterate, 18 for individuals with 1 to 7 years of education, and 26 for individuals with 8 years or more of Education^(20)^. There was no exclusion.

### Stages of the study

#### 1) Development of interactive gerontechnology

The first step towards the creation of gerontechnology was the use of the study by Lima and collaborators^(10)^, who developed a three-dimensional technology for the prevention of falls in the elderly at home, but with limitations in terms of size, interactivity, and number of rooms. Based on these limitations, the study followed with the construction of the interactive educational scale model.

The study developed two prototypes until the final version of gerontechnology. Prototypes are all representations that simulate some aspect of the product or idea to be developed^(21)^. For the initial prototype, we counted on the participation of an architect in the elaboration of the project that represented a home in a ground plan, considering the survey of the literature and the recommendations arising from the situational diagnosis.

AutoCAD^®^ software created the two-dimensional drawing of the floor plan, which divided the rooms and distributed the specifics corresponding to a real house, with partitions and furniture, seeking for a realistic representativeness. The floor plan was enlarged and modeled three-dimensionally in a virtual scale model with the use of SketchUp^®^ software, version 2018 for Windows.

#### 2) Evaluation of interactive gerontechnology by expert judges

In the evaluation stage with the judges, the first contact occurred through the channel offered by Lattes. The message requested the email address to formalize the invitation and asked about the possibility of participating in the survey. Researchers sent the Informed Consent Form (ICF) and the invitation letter by email. Thus, upon acceptance, participants received: an electronic link to access a video containing the presentation of Prototype 1 (P1) with a duration of 8 min 9 sec available on the video-sharing platform YouTube; and the evaluation instrument with questions related to the objectives of the scale model, general and specific organization, usability, applicability and functionality, made available by a cloud research development company called SurveyMonkey^®^.

The expert judges completed an instrument composed of 38 items divided into five blocks, of which five corresponded to the objectives of the scale model, 23 referred to the general and specific organization regarding usability; four to the applicability, and two to functionality, with the following options proposed on a Likert scale: 1 = disagree; 2 = Partially disagree; 3 = neither disagree nor agree; 4 = partially agree, and 5 = agree. In addition, the evaluation instrument had two sections: an initial one with questions related to expertise and practice related to fall prevention and accessibility; and another containing a specific field aimed at obtaining suggestions and opinions on the aforementioned blocks.

#### 3) Evaluation of interactive gerontechnology by the target audience

The study used the theoretical framework of the Health Promotion Model (MPS), by Nola Pender, to subsidize the use of the interactive scale model, which has three components: (1) individual characteristics and experiences refer to the previous behavior that must be changed; (2) feelings and knowledge about the specific behavior are the perceptions of benefits for action and perception of barriers to action, and (3) desirable Health Promotion Behavior refers to the health promotion behavior that is desired to achieve^(22)^.

In the stage of evaluation of educational technology by the target audience, the P2 prototype was presented individually to the elderly with questions guided by the MPS, namely: (1) individual experiences: does this scale model resemble your home or the home of someone you know? Are this furniture and accessories present in your home? Have you fallen in the last year? If so, where did the fall occur? (2) Specific behavior: could you point out in each room what would facilitate the occurrence of falls? What can hamper the reduction of risks in your home? How can these changes help avoid risks? (3) Expected outcome: what behaviors would you adopt to decrease the risk of falls?

After the individual presentation, the study moved to the second stage that evaluated: 1) appearance; 2) usability; 3) motivational characteristics for learning, and 4) satisfaction. In addition, there was a field for suggestions. The instrument used quantified the level of agreement with the Likert scale: 1 = disagree; 2 = partially disagree; 3 = neither disagree nor agree; 4 = partially agree, and 5 = agree.

### Analysis of results and statistics

The identification and evaluation data of the judges and the elderly were compiled and analyzed by IBM^®^ SPSS^®^ Statistics software for Windows, Version 23.0, through descriptive statistics and confidence interval. An Agreement Index (AI) of 80% was adopted between the evaluations of the judges and the elderly and the Wilcoxon test was applied with a significance level of 5%.

## RESULTS

### 1) Development of interactive three-dimensional gerontechnology of scale model-type

**Figure 1:**
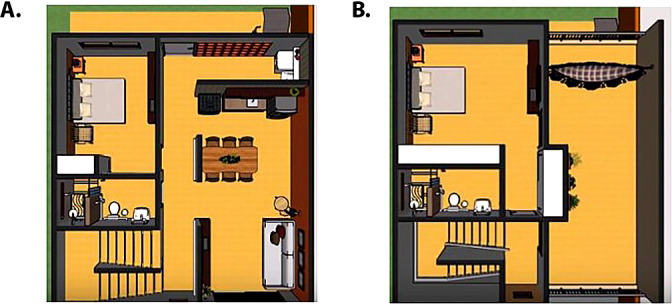
Representation of the ground floor (A) and the first floor (B) in a three-dimensional project of gerontechnology for the prevention of falls in the elderly

The scale model has an area of 60 cm x 55.6 cm, with a scale of 1:15 - reasonable dimensions for an adequate demonstration of the risks of falls at home. The scale model represented dimensions of 9 m x 8.35 m, considering the average of two residents and the minimum amount of space for each one. It used lightweight and resistant materials: a combination of expanded polyvinyl chloride (PVC), Medium Density Fiberboard (MDF), and acrylic (Figure 1).

The materials were painted with special automotive paint of prolonged duration and strength.

### 2) Evaluation of interactive gerontechnology by expert judges

Fifty-four specialists evaluated the scale model, whose ages ranged from 25 to 70 years (M = 41.02; SD = +11.4 years). Among these, 43 (79.6%) were women, and 11 (20.4%), were men; 30 (55.5%) were nurses, 10 (18.5%) were physiotherapists, 6 (11.1%) were architects, 4 (7.4%) occupational therapists, 3 (5.5%) physicians, and 1 (1.8%) physical educator; 46 (85.2%) were specialists, 43 (80%) masters, 33 (61.1%)%) PhDs, and 7 (13%) postdocs. Three professors (5.6%) from a Nursing higher education institution in Portugal participated in the study.

As for the area of activity, 26 (48.1%) worked in teaching, 17 (31.5%) in assistance/work with consumers, and 11 (20.4%) exclusively in scientific research. Concerning scientific production, 47 (87%) had published articles on at least one of the following topics: education; Geriatrics; Gerontology; creation and evaluation of educational materials; accessibility and adaptation of spaces for the elderly and people with disabilities; falls and fall prevention. Regarding experience, 36 (66.6%) reported experience with the evaluation and/or validation of educational materials in health.

**Figure 2:**
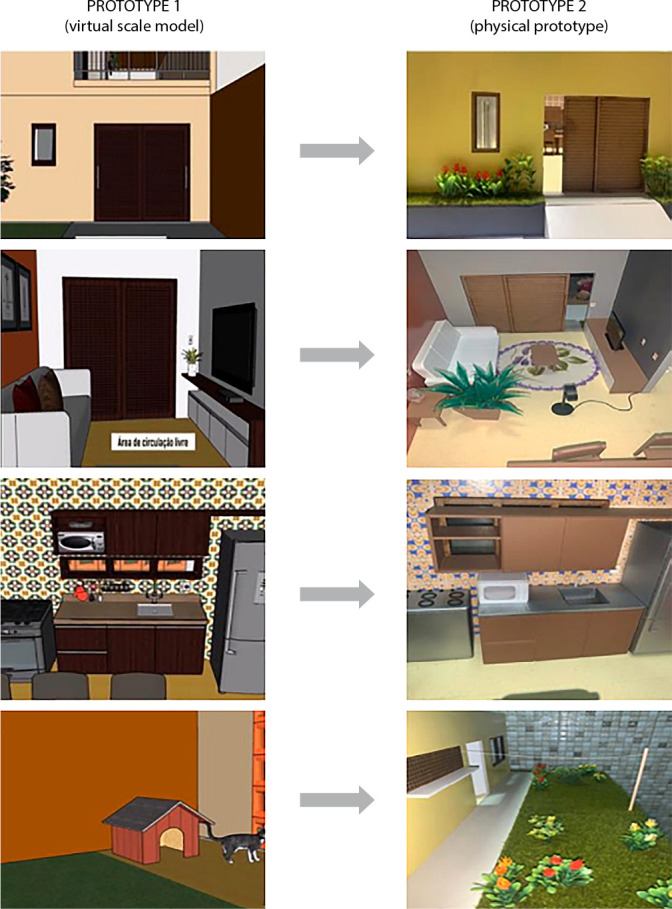
Modifications conducted based on the suggestions made by the experts in the evaluation of Prototype 1 (P1) of the scale model

During the evaluation, the judges made suggestions that were accepted for the modifications of the interactive educational scale model. These suggestions were about the entrance (inclusion of ramp and gardening), living room (inclusion of removable items: coffee table, rugs, vases, scattered wires and cables, and fan), dining room/kitchen (reduction of microwave height), and backyard (gardening, shelf, clothesline, and pets) (Figure 2).

**Figure 3:**
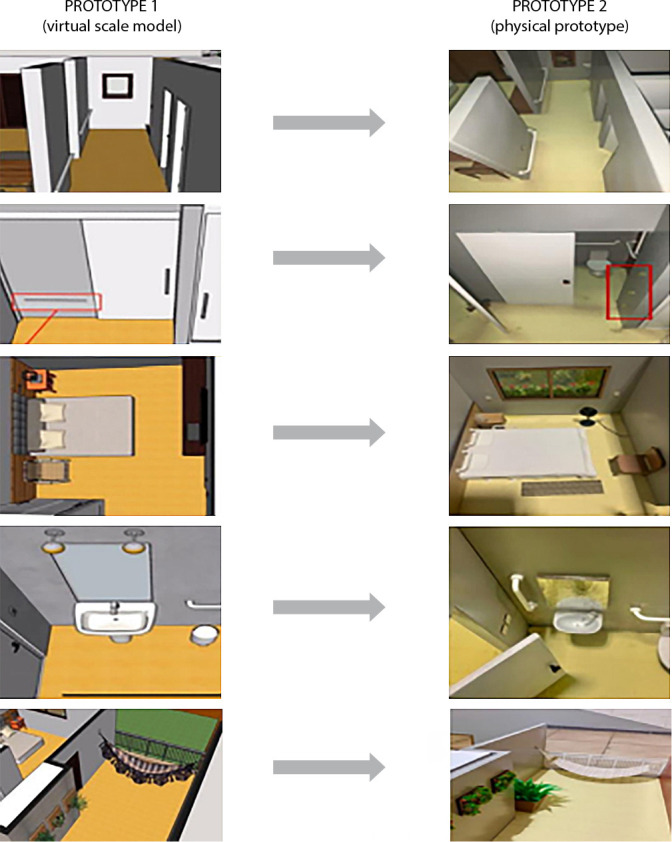
Modifications conducted based on the suggestions made by the experts in the evaluation of the Prototype 1 (P1) of the scale model

Judges also suggested modifications for the corridor (support bars), bedroom (night lighting, inclusion of carpets, wires, fans, furniture of less robustness, and bedspreads touching the ground), bathroom (added support bar next to the sink), staircase and balcony (inclusion of chair, plant jars, removal of the “fringes” of the hammock). In addition, they made general suggestions, such as the elaboration of a manual and the inclusion of characters with potential risks of falling to make the scale model more attractive (Figure 3)

**Figure 4:**
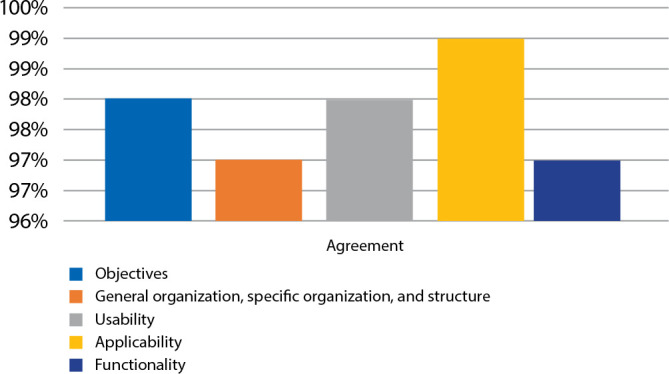
Agreement index regarding the aspects evaluated by the experts

The scale model was considered valid as educational gerontechnology in the prevention of falls in the elderly, with an average Agreement Index of 97%. The Wilcoxon test, which establishes the position parameter, found that the *p*-value statistic was less than 0.001 in all items, whose AIs were greater than 0.80 (Figure 4).

### 3) Evaluation by the target audience

Of the 30 seniors who evaluated the scale model, 25 (83.3%) were women, and 5 (16.6%) were men, with ages ranging between 63 and 91 years (mean ± standard deviation: 73.2 ± 6.8) and mean schooling of 6.1 (±5.2) years of study. Concerning income, the average individual monthly was 1.2 (±1.1) minimum wage.

Regarding falls, 22 (73.3%) reported being afraid of falling, and 17 (56.6%) had fallen in the last 12 months; 24 (80%) were of their height, 6 (20%) fell from the hammock, and 5 (16.6%) from the chair. Regarding the places of falls, 17 (56.6%) mentioned having fallen on the street, 8 (26.6%) in the backyard, 7 (23.3%) on the sidewalk, 5 (16.6%) in the bathroom, 5 (16.6%) in the bedroom and 5 (16.6%) in the living room.

In the use of the scale model, the elderly suggested the inclusion of toys scattered around the house, as it is common for the elderly to live with grandchildren, which is a cause of falls. Another suggestion was to have a kind of support next to the bed or at the headboard so that the elderly can hold themselves when they get up.

**Figure 5:**
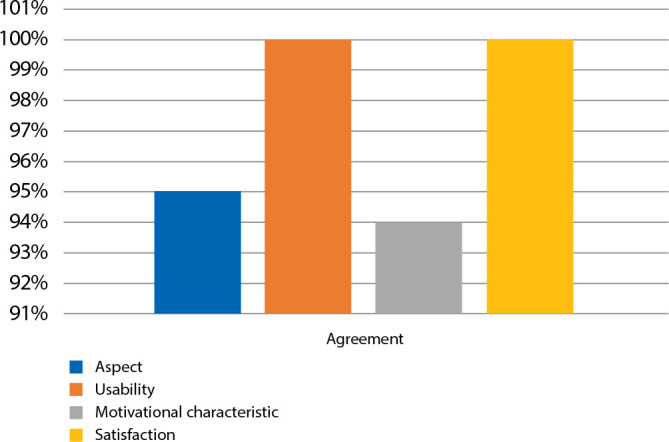
Agreement index regarding the aspects evaluated by the elderly of the community

The scale model was considered valid by the elderly public, with an average AI of 97%. The Wilcoxon test, which establishes the position parameter, found that the *p*-value statistic was less than 0.001 in all items, whose AIs were higher than 0.80 (Figure 5).

Participants considered the representation and interactive approach of the scale model as attractive and unprecedented, and it helped them to remain attentive during the practice of health education and the presentation of technology. Seniors were able to score and demonstrate risk factors, as well as report behaviors necessary to promote health and prevent falls at home.

## DISCUSSION

The use of prototypes in gerontechnology development study allows preliminary versions, which can be viewed and evaluated during the creation process. The use of prototypes is an efficient and economic strategy since it allows the identification of possible failures through tests and evaluations, ensuring correction before the final version of the product^(23)^.

The choice of high fidelity prototype of the final version was fundamental for the adequate visualization and contemplation of the items to be corrected or included. Studies indicate that the adoption of a functional prototype is essential for improvements in the user interface and optimization of application performance^(24-26)^.

In the evaluation process of gerontechnology of scale mode-type by expert judges, the choice of individuals from different backgrounds stands out, which favored a genuine evaluation regarding the prevention of accidents due to falls. Studies show that the home is the principal place where falls occur in the elderly, and this outcome can be avoided through prevention and health promotion measures. It is necessary to look at the adequacy of the environment to provide increased safety and quality of life for the elderly^(2,27-29)^.

The judges’ and the elderly suggestions were accepted and are in line with the evidence found in a systematic review that investigated the role of home modification interventions in reducing the rate and risk of falls among the elderly^(30)^. The most suitable conditions for preventing falls are correct lighting, non-slip flooring, organization of the environment, the absence of carpets, and a bulkhead for seating during bathing. They also indicate that non-slip material can be used on the steps and grab bars to facilitate access to the ladder^(31)^.

Guidance for some home modifications, such as improving the organization of the environment, can effectively prevent the incidence of falls. All these findings were included in gerontechnology aiming to achieve multiple approaches and demonstrations with the elderly^(4)^. Authors^(32)^ conclude that the fall of the elderly is associated with steps, unevenness, carpet, pets, and objects on the floor. It requires preventive measures to be adopted by health professionals, family and community.

A study with the elderly with a history of falls based on an interactive and personalized approach showed that the public tends to positively receive guidance and increase engagement in prevention measures when these represent the individual reality^(33)^.

The use of the Health Promotion Model helps integrate Nursing with behavioral science, identifying factors that influence health behaviors. The application of this model satisfactorily guided the exploration of aspects related to the motivation or demotivation of seniors in engaging in behaviors that promote healthy aging^(34)^.

HPM considers individual characteristics, benefits, and barriers to the adoption of healthy behaviors. A study^(10)^ reinforces that the use of the scale model for the prevention of falls in the elderly promotes dialogue and the exchange of experiences, which favors health promotion and provides a critical-reflective attitude of the elderly concerning their co-responsibility in the prevention of falls.

In this sense, studies highlight the importance of guidelines for the prevention of falls in the elderly emphasizing extrinsic factors, such as home adaptation, to ensure a safe environment. They also focus on the social factor of the elderly that influences the occurrence of falls. The measures consist of guiding the elderly and the family about the risks and consequences through health promotion so that the risk factors are corrected or minimized^(29,35)^.

Most reported not having received prior guidance on fall prevention, and of those who did, the nurse was the most cited professional in this practice. Among the guidelines offered, the main one was regarding the carpet removal and installation of non-slip floors. This study emphasizes the knowledge of seniors about falls and recommends that health professionals, particularly nurses, evaluate their risk perception regarding risk factors in daily life and advise on prevention measures^(36)^.

The elderly’s suggestions during the development of gerontechnology were accepted, emphasizing the inclusion of the demonstration of toys around the house. This fact is relevant since the number of trigenerational arrangements has grown in recent decades, with a higher number of co residents and greater difficulties in maintaining the circulation space free of obstacles and organized^(37-38)^.

The educational gerontechnology developed in this study, although it does not represent the totality of the residences of the various Brazilian elderly, constitutes an option of multiple approaches, with the possibility of approaching the house integrally or rooms separately and demonstrating extrinsic risks of falls or adaptations recommended in the literature. In addition, it fills a gap previously perceived in the scientific field with the provision of an educational resource option for fall prevention in three dimensions, enabling more realistic visualization than the two-dimensional offered in widely distributed printed materials.

The scale model produced was designed to facilitate dialogue between the target audience and professionals and simulate everyday situations of risks that the elderly face during the execution of daily life activities. Therefore, it can be used in health education practices on fall prevention with the health professionals’ support, helping the elderly to identify and understand the risks linked to this condition and, consequently, adopt a health-promoting behavior, as well as awaken reflections on the subject. A multidisciplinary team can use it, especially in Primary Health Care, where the health team is closer to the population to adapt the scale model according to the individual’s reality. However, a limitation of the adoption of this educational gerontechnology in health education practices is the high costs of its manufacture.

Strict compliance with the methodological stages with clarity in the elaboration phases was crucial to guarantee a robust final construct. In this sense, a study on the application of an educational scale model for the prevention of falls in the elderly in Japan did not demonstrate transparency about the stages of development, and it does not allow other researchers to replicate the study^(39)^.

Results showed that the scale model built in this research was considered suitable for the educational three-dimensional gerontechnology for fall prevention in the elderly. The positive evaluation was due to the use of an adequate evaluation method based on current literature, the hiring of qualified professionals for the creation and execution of the project, and the use of reference programs for the development of the preliminary prototype.

In this study, the participation of more than a hundred individuals, involving the target audience, judges, and specialized professionals, was indispensable for obtaining promising results in the development of a gerontechnology that can be replicated in other scenarios and even in other aspects of health.

### Study limitations

Although the scale model has been innovative in terms of its reduced dimensions and the use of lighter materials, it still has a considerable weight and needs adequate space, albeit minimal, for its installation and use, which can be a challenge. There is the possibility of transforming interactive gerontechnology into virtual reality, providing users with immersion in this reality.

### Contributions to the fields of Nursing, Health, or Public Policy

The use of interactive gerontechnology has the potential to contribute to health practices in various contexts, with nursing professionals and other areas that address Gerontological care. It can assist in educational activities and appointments to complement the stimulation of active aging and individualized care, which result in the reduction and prevention of conditions and diseases that may affect elderly victims of falls.

## CONCLUSIONS

The study considered the interactive educational gerontechnology of the scale model-type valid to be used as a strategy for health promotion and prevention of falls at home in the elderly of the community. It is an innovative option to enable the practice of health education with the elderly population to prevent falls and their consequences on the population.
